# A Novel *Bmr6* Allele (*bmr34*) Confers the Brown Midrib Phenotype Without Biomass Penalty in Sorghum

**DOI:** 10.3390/plants15111630

**Published:** 2026-05-26

**Authors:** Lei Li, Yongfei Wang, Qi Shen, Wenmiao Tu, Kai Wang, Die Hu, Lihua Wang, Yi-Hong Wang, Jieqin Li

**Affiliations:** 1 College of Agriculture, Anhui Science and Technology University, Fengyang 233100, China; 13275503216@163.com (L.L.); 11962@ahstu.edu.cn (Y.W.);; 2 Anhui Provincial International Cooperative Research Center for Forage Biological Breeding, Fengyang 233100, China; 3 Department of Biology, University of Louisiana at Lafayette, Lafayette, LA 70504, USA

**Keywords:** *Sorghum bicolor*, brown midrib, *bmr34*, lignin, KASP tag

## Abstract

Brown midrib (*bmr*) mutants are frequently associated with unfavorable agronomic traits. In this study, we identified a novel brown midrib mutant, *bmr34*, which exhibited distinct brown coloration in roots, stems, and leaf midribs. Although most classic *bmr* mutants show undesirable agronomic performance, this mutant displays altered lignin accumulation and has important potential for forage quality and biomass utilization, providing a key genetic resource for lignin regulatory research in sorghum. Compared to the wild-type, *bmr34* showed no significant differences in five major agronomic traits; however, lignin content was significantly reduced. Bulked segregant analysis (BSA) using an F_2_ population derived from a cross between *bmr34* and the wild type Tx623 mapped the candidate region to chromosome 4. Further sequencing analysis identified a single nucleotide substitution (C → T; reverse strand G → A) at position 5,731,348 within the 5′ splice site of the third intron of *Sobic.004G071000* in the mapping interval. KASP marker analysis demonstrated complete co-segregation between the mutation site and the *bmr* phenotype. Sequence analysis also revealed that this G → A substitution resulted in aberrant splicing and a 33-bp insertion in the third exon, which introduced a premature stop codon. Notably, the normally spliced transcript still accounted for approximately 36.2% of total transcripts in *bmr34*, indicating partial retention of wild-type transcript processing. These results demonstrate that *bmr34* represents a novel weak allele of *Bmr6*, providing new insights into splice-site mutations and their contribution to lignin biosynthesis regulation in sorghum.

## 1. Introduction

Sorghum [*Sorghum bicolor* (L.) Moench] ranks as the fifth most important cereal crop worldwide [1] and is now widely cultivated across the globe. It not only serves as a high-quality forage raw material for animal husbandry but can also be converted into clean energy such as ethanol, in addition to being used in miscellaneous grain food processing, thus conferring both economic and ecological benefits. Moreover, sorghum exhibits remarkable tolerance to a range of abiotic stresses. It is highly drought tolerant [2], adaptable to poor soils [3], and flood tolerant. It also shows strong resilience to salinity–alkalinity stress and high temperatures [4].

Sorghum leaf midrib is typically white [5], whereas plants with the *brown midrib* (*bmr*) mutation exhibit brownish-gray or brownish-red veins and stems [6]. This mutation was first identified in maize in 1924 [7]. Subsequently, similar mutations were identified in sorghum and pearl millet [8]. To date, 13 *bm* mutants have been reported in maize [9], all representing recessive natural variants. These mutations can be grouped into six alleles [10], and all related genes have been cloned. *Brown midrib* mutations in sorghum originated from chemical mutagenesis and have been categorized into eight genetic loci to date [11]. However, only the *Bmr2*, *Bmr6*, and *Bmr12* genes have been cloned and functionally characterized. *Bmr2*, located on chromosome 2, encodes a coenzyme A ligase (4CL) [12]. Mutations in *bmr2* cause a significant reduction or complete loss of enzyme activity [13], thereby preventing the effective activation of hydroxycinnamic acids and ultimately reducing lignin content in plants [14]. *Bmr6*, which is located on chromosome 4 [15], encodes an alcohol dehydrogenase (CAD) [16]. Mutations in this gene severely inhibit the catalytic activity of CAD, blocking the conversion of cinnamaldehyde to cinnamyl alcohol and consequently impairing the synthesis of lignin monomers [17]. *Bmr12*, located on chromosome 7, encodes caffeic acid O-methyltransferase (COMT) [18]. Loss-of-function mutations in *Bmr12* result in reduced or abolished COMT activity, disrupting normal methylation reactions and specifically inhibiting the synthesis of syringyl (S)-lignin monomers. These three genes code for important enzymes involved in the lignin monomer synthesis pathway. Previous studies have shown that *bmr* mutants exhibit low lignin content, accompanied by increased levels of digestible hemicellulose and cellulose [19], thereby significantly enhancing forage digestibility in ruminants [20].

It is thus generally believed that darker midrib color correlates with superior forage quality [21]. Consequently, brown midrib sorghum is considered a forage resource with high nutritional value. Oliver et al. [22] demonstrated that feeding dairy cows with brown midrib mutant sorghum resulted in significantly improved performance compared to conventional sorghum. However, although brown midrib mutations effectively reduce lignin content, they may also negatively affect key agronomic traits, including plant height and biomass [23]. Wang et al. [24] conducted comparative analyses across five different sorghum genetic backgrounds and found that the *bmr6* and *bmr12* genes exerted distinct effects on fresh weight. Specifically, in the Kansas Collier and Early Hegari-Sart genetic backgrounds, both *bmr6* and *bmr12* significantly reduced biomass yield. Therefore, further screening for brown midrib mutants that do not compromise biomass production, along with cloning and functional characterization of the underlying genes, is essential for the development of high-quality forage sorghum.

In this study, a novel brown midrib mutant, *bmr34,* was identified from the EMS-mutagenized sorghum Tx623 population. Compared to the wild-type (WT), *bmr34* showed no significant differences in plant height, tiller number, or biomass. Bulked Segregant Analysis (BSA), Kompetitive Allele-Specific PCR (KASP), and gene cloning analysis identified *bmr34* as a new allelic variant of *Bmr6*.

## 2. Materials and Methods

### 2.1. Materials

Wild-type BTx623 and *bmr34* were provided by the International Joint Research Center of Forage Bio-Breeding in Anhui Province. Sorghum materials were planted in the experimental field under natural conditions, with conventional field management including watering, fertilization, and pest control.

### 2.2. Research Methods

#### 2.2.1. Observation of Midrib and Stem Sections in WT and *bmr34*

At the 7-leaf stage, midribs of WT and *bmr34* were cut into 100 μm sections by an ultramicrotome (EMUC7, Leica, Wetzlar, Germany). Wiesner staining was performed by incubating sections in 1% phloroglucinol in ethanol: water (7:3) with 30% HCl. Staining sections were observed using a stereomicroscope (YSZ710T, YUESCOPE, Hefei, China).

#### 2.2.2. Measurement of Traits of Wild Type and *bmr34*

At the heading stage, five agronomic traits were measured: plant height, stem diameter, effective tiller number, fresh weight per plant, and dry weight per plant. Dry weight per plant: After determining fresh weight, plants were incubated at 105 °C for 1 h. Then, they were dried for 48 h until the sample weight remained constant (difference between two consecutive measurements ≤0.02 g). Lignin content was determined using the acetyl bromide method [25].

#### 2.2.3. DNA and RNA Extraction

DNA was extracted with the magnetic bead-based kit (DP151130, TianGen Biochemical Technology Co., Ltd., Beijing, China). for parents and BSA pools, following the manufacturer’s protocol. Total RNA extraction from leaves followed the protocol of the RNAprep Pure Plant Total RNA Extraction Kit (B0116A, Tiangen Biotech (Beijing) Co., Ltd., Beijing, China).

#### 2.2.4. Bulked Segregant Analysis (BSA)

The F_2_ population was derived from a cross between *bmr34* mutants and WT. BSA was performed as follows. Briefly, 30 individuals with white midrib and 30 individuals with brown midrib were selected from the F_2_ population. Genomic DNA was extracted from the parental lines and each selected F_2_ individual. Equal quantities of DNA from F_2_ individuals were pooled separately to construct a wild-type (white midrib) DNA pool and a mutant (brown midrib) DNA pool. The mutant and two pools were sent to BGI for second-generation sequencing. QTL-seq software (qtlseq 2.1.2) [26] was used to map the *bmr34* gene using Tx623 V3.0.1 as the reference genome. SNPs and indels were annotated using ANNOVAR (2019Oct24) [27] in the target region.

#### 2.2.5. KASP

PCR amplification was performed using a 10 μL PCR reaction volume consisting of 2 μL of genomic DNA template (30 ng/μL), 0.14 μL of primer mix (FAM: VIC: forward primer: ddH_2_O = 6:6:18:30, *v*/*v*), 5 μL of KASP Mix, and 2.86 μL of deionized water. The PCR program was set as follows: pre-denaturation at 95 °C for 10 min; followed by 10 cycles of touchdown PCR (denaturation at 95 °C for 15 s, annealing at 65–55 °C for 15 s with a 1 °C decrease per cycle); then 42 cycles (denaturation at 95 °C for 30 s, extension at 57 °C for 60 s); and a final fluorescence reading at 30 °C for 30 s. Fluorescent PCR products were detected using QuantStudio^TM^ 7 (4485701, ThermoFisher Scientific, Waltham, MA, USA).

#### 2.2.6. Cloning of Mutation Site

Cloning primers were designed with Primer Premier 5.0 software based on the mutation site (Table S1). PCR amplification was performed in a 30 μL reaction system, which contained 3 μL of cDNA, 5 μL of primer mixture (F1:R1:ddH_2_O = 1:1:48, *v*/*v*), 0.6 μL of KOD DNA polymerase, 6 μL of dNTPs, and 15.4 μL of 2× KOD buffer. PCR thermocycling was set as follows: pre-denaturation at 95 °C for 7 min, followed by 36 cycles of denaturation at 95 °C for 30 s, annealing at 56 °C for 30 s, and extension at 72 °C for 40 s. After amplification, PCR products were run on a 2% agar gel at 120 V. The amplification band was extracted from the gel using a Gel Extraction Kit (UE-GX-250, Uelandy Biotech Co., Ltd., Suzhou, China). The extracted bands were linked with a cloning vector using the Clone Enzyme 5 min TA/Blunt-Zero Cloning Kit (C601-01, Vazyme Biotech Co., Ltd., Nanjing, China). Sanger sequencing was conducted by (Sangon Biotech Co., Ltd., Shanghai, China).

### 2.3. Data Statistics

The data were analyzed using GraphPad Prism 5 software.

## 3. Results and Analysis

### 3.1. Phenotypic Observation of the bmr34

**Figure 1:**
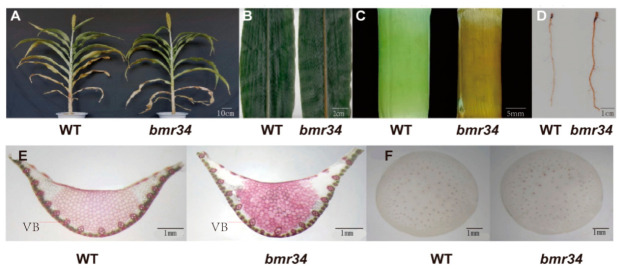
Phenotypes and sections of WT and *bmr34* mutant. (**A**): At heading stage, scale bar = 10 cm. (**B**): Midribs in the second-to-last leaf at jointing stage. (**C**): Fourth internode of stems at the 7-leaf stage. (**D**): Root systems. (**E**): Leaf midrib sections at heading stage, VB represents the vascular bundle, scale bar = 1 mm. (**F**): Stem sections at heading stage, scale bar = 1 mm.

Compared to WT, *bmr34* showed no obvious difference in plant morphology (Figure 1A), other than a distinctly visible brown coloration in midrib, stem, and roots from the six-leaf stage through all subsequent growth stages (Figure 1B–D). The results demonstrate that the *bmr34* mutant is a typical sorghum *bmr* mutant, characterized by a stable brown phenotype that extends beyond leaf midribs to multiple tissues.

To investigate the effects of the *bmr34* mutation on the structure and composition of leaf midribs and stems, *bmr34* and WT were cross-sectioned, which showed no obvious difference in vascular bundle arrangement, tracheary element morphology, or cell organization of the cortex and pith between them (Figure 1E,F). However, the staining intensity around the vascular bundles and xylem of the mutant was significantly stronger than that in WT plant midribs and stems (Figure 1E,F). This indicates that the *bmr34* mutation does not affect the fundamental tissue architecture but decreases the lignin content in leaf midribs and stems.

### 3.2. Comparison of Agronomic Traits Between WT and bmr34

**Figure 2:**
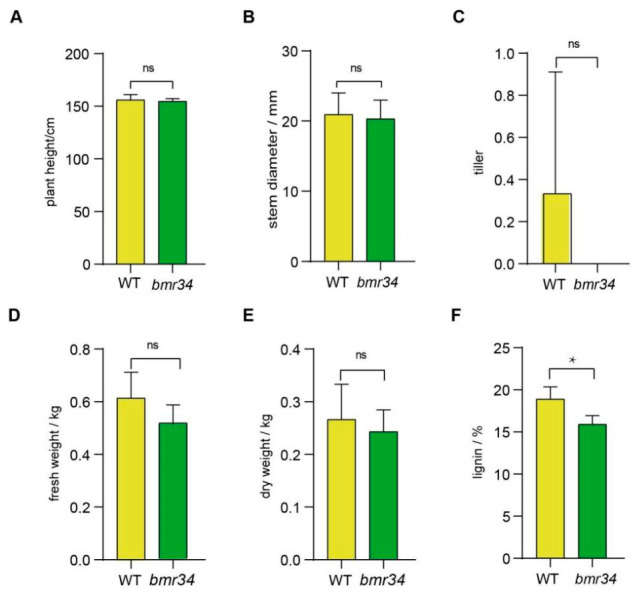
Agronomic traits and lignin content of WT and *bmr34* mutants. (**A**): Plant height; (**B**): Stem diameter; (**C**): Tillering; (**D**): Fresh weight; (**E**): Dry weight; (**F**): Lignin content. Error bars represent standard deviation from three replicates. Data points indicate means. ns denotes no significant difference; * denotes significance at *p* < 0.05.

To further investigate phenotypic differences, we compared five agronomic traits between WT and *bmr34*. The results showed that there were no significant differences in plant height, tiller number, or stem diameter between WT and *bmr34* (Figure 2A–C). Subsequently, we also did not observe a significant difference in biomass (fresh weight per plant) and dry weight per plant between them (Figure 2D,E). However, compared to WT, the lignin content of *bmr34* was reduced by 16%. Taken together, the *bmr34* mutant has lower lignin content and a biomass yield comparable to that of the WT.

### 3.3. Mapping and Candidate Gene of bmr34

To map the gene underlying the *bmr34* trait, an F_2_ segregating population was developed by crossing the *bmr34* mutant with the WT. All F_1_ progenies exhibited a non-brown-midrib phenotype. Chi-square test results revealed that the segregation ratio of non-brown midrib to brown midrib phenotypes in the F_2_ population conformed to the classic Mendelian 3:1 ratio (Table S2), demonstrating that the *bmr34* trait is governed by a single recessive nuclear gene. Bulked segregant analysis (BSA) was adopted for genetic mapping of the target gene in this study. Detailed BSA sequencing data for the brown midrib bulk and non-brown midrib bulk are provided in Table S3. The clean bases for *bmr34* and the two pools are more than 24G, 30 times the size of the sorghum genome. Q20 (representing the percentage of bases with a sequencing accuracy of more than 99%) was over 95%, and the mapping rate of clean reads against the reference genome also exceeded 95% for all samples. These results suggest that the sequencing quality met the needs for BSA, thereby excluding experimental biases induced by insufficient sequencing data or low sequencing quality.

**Figure 3:**
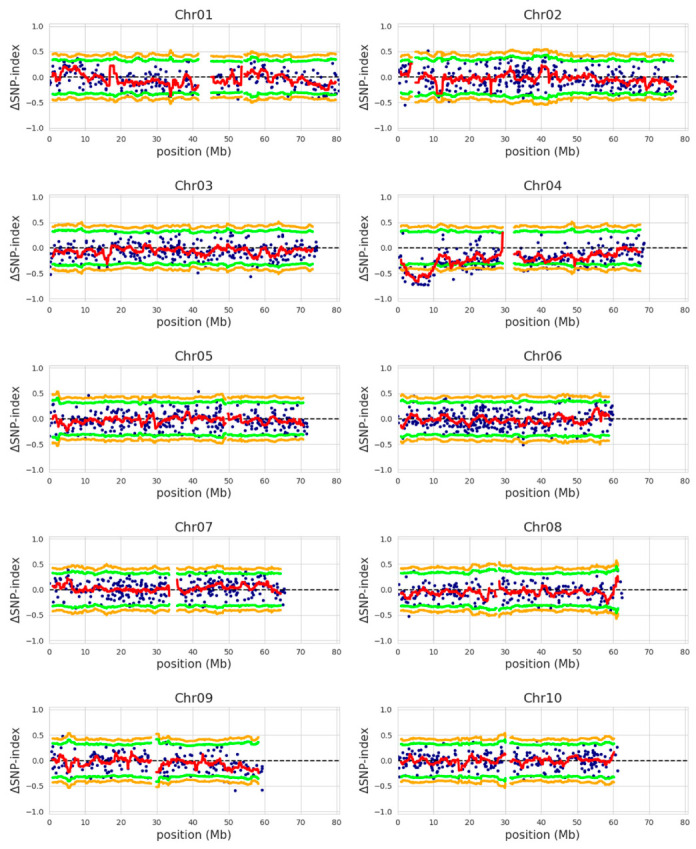
BSA linkage region plot: The distribution of ΔSNP-index values from chromosome 1 (Chr01) to chromosome 10 (Chr10) showed that the ΔSNP-index values in the 0 Mb–10 Mb range of chromosome 4 were significantly lower than the predicted range. Red dots represent ΔSNP-index values; the green line indicates the 95% confidence interval threshold, and the yellow line denotes the 99% confidence interval threshold.

**Figure 4:**
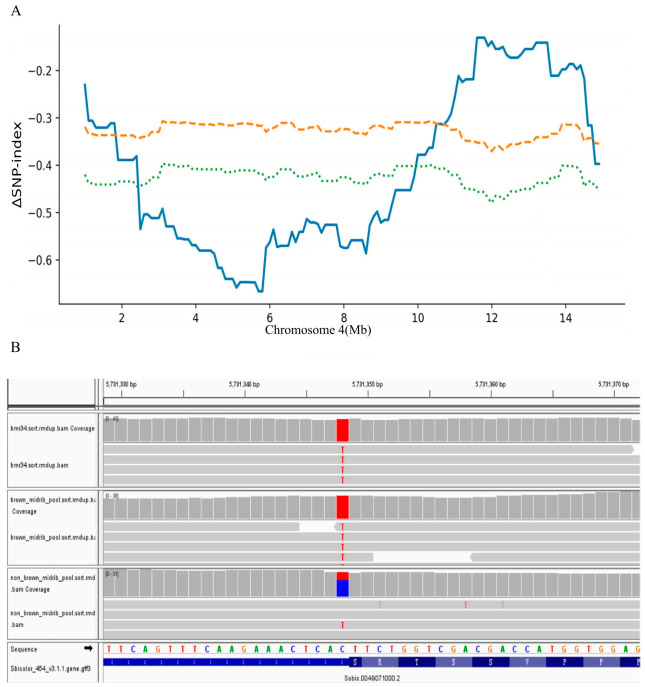
Localization of the *bmr34* gene in brown midvein. (**A**): Sliding window analysis of ΔSNP-index along chromosome 4 of *Sorghum bicolor*. The blue line represents the ΔSNP-index value; the green dashed line indicates the 95% confidence threshold, and the yellow dashed line denotes the 99% confidence threshold. (**B**): Multi-track visualization of genomic annotations for the candidate region in the sorghum genome.

The sequencing results showed that *bmr34* was mapped on chromosome 4. The ΔSNP-index in this interval showed a significant overall decrease and fell below the significance threshold line, indicating that the candidate gene controlling the brown midrib trait is located within the physical interval of 0–10 Mb on chromosome 4 (Figure 3). Further analysis confirmed that *bmr34* was in the region of 2.5–9.9M based on a 0.99 ΔSNP-index threshold value (Figure 4A). There were 22 SNPs and 35 indels in the region. Further annotation showed that there were no variants in the exons of any genes (Table S4). Only one variant (position 5731348) from C to T (reverse G to A) was identified at the splicing site of exon 3 of *Sobic.004G071000* (Figure 4B), which encodes cinnamaldehyde dehydrogenase in the biosynthesis of the lignin pathway. A previous study already identified *Sobic.004G071000* as *Bmr6* [28].

### 3.4. Development and Validation of KASP Markers

**Figure 5:**
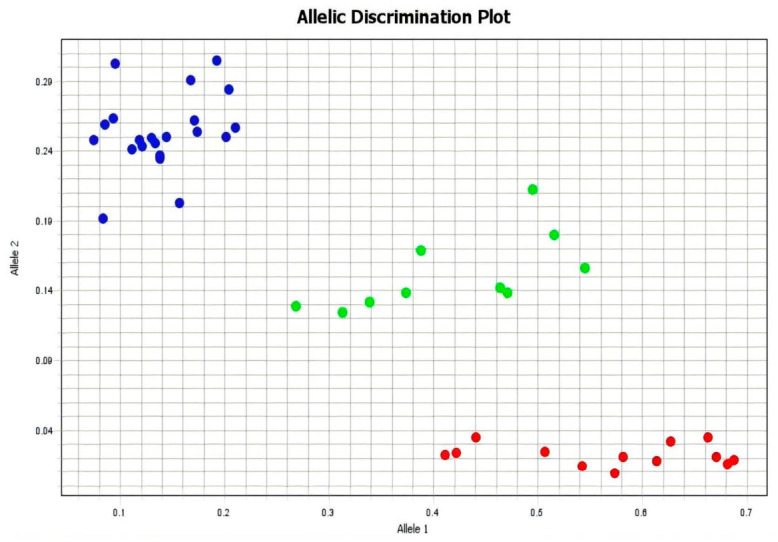
Genotyping results of the *bmr34* segregating population. Genotyping results in the F_2_ population: red dots indicate paternal genotype, blue dots indicate maternal genotype, and green dots indicate heterozygous genotype.

To further validate the candidate gene, a KASP marker was developed based on the variant at the 5′ splice site. The KASP assay clearly discriminated the maternal/paternal parents and heterozygous progeny (Figure 5). Genotype–phenotype association analysis was conducted using 44 individuals from the F_2_ population. All 13 paternal homozygotes exhibited the brown midrib phenotype, whereas the 10 heterozygotes and 21 maternal homozygotes displayed the non–brown midrib phenotype. The 10 heterozygous individuals were further selfed, and all progeny populations segregated for the brown midrib trait. These results demonstrate complete cosegregation between the KASP marker and the brown midrib phenotype.

### 3.5. Gene Cloning for bmr34

**Figure 6:**
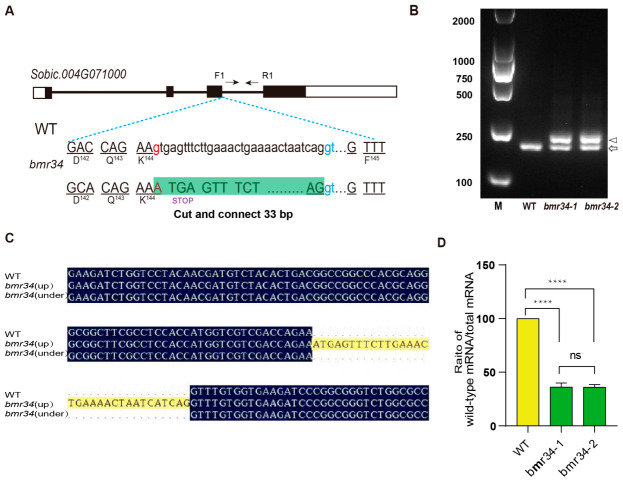
Gene localization and mutation types of the brown midrib *bmr34* gene. (**A**): Analysis of the *bmr34* mutant site. Compared to the wild type, *bmr34* exhibits a 33 bp (insertion). F1 and R1: detection primer positions; (**B**): Electrophoresis of *WT* and *bmr34* mutant on a 2% agarose gel. The arrow points to the mutant band (209 bp), while the arrowhead indicates the wild-type band (176 bp). (**C**): Sequence comparison of WT and *bmr34* mutant cDNAs. (**D**): Grayscale value distribution map for WT and *bmr34* mutant. The y-axis represents the ratio of wild-type transcript to total *Sobic.004G071000* mRNA. Data are presented as the mean ± standard deviation (SD) of four biological replicates (*n* = 4). Statistical significance was determined by one-way ANOVA followed by Tukey’s multiple comparison test. **** *p* < 0.0001; ns, not significant.

To characterize the molecular basis of the *bmr34* mutation, we analyzed the full-length transcript of the candidate gene *Sobic.004G071000* in both the wild type (WT) and *bmr34* mutant. Gene structure and sequence alignment revealed that a 33-bp sequence was abnormally retained and inserted into the mature transcript of *bmr34* due to altered splicing at the third exon–intron junction (Figure 6A). Although the length of this insertion is a multiple of three and does not cause a frameshift, the inserted sequence contains an in-frame premature stop codon (TGA) immediately downstream of the insertion site. Consequently, translation is prematurely terminated, leading to a predicted truncated protein that lacks the downstream conserved functional domains. This indicates that the aberrant transcript in *bmr34* encodes a nonfunctional protein, which likely accounts for the brown midrib phenotype in the mutant. Primers F1 and R1 were designed to amplify the target region containing the insertion site, confirming the presence of the 33-bp insertion in the *bmr34* transcript.

To confirm the effect of the 5′ splice-site variant, a specific primer was designed to detect splicing alterations at the RNA level. RT–PCR analysis showed that the wild-type (WT) produced a single band corresponding to the normally spliced transcript (open arrow), whereas *bmr34* cDNA yielded two bands. One band was identical in size to the WT product, while the other was larger (solid arrow) (Figure 6B). Sequencing analysis revealed that the WT-sized band had the same sequence as the wild-type transcript, whereas the larger band contained a 33-bp insertion in the third exon of *bmr34* (Figure 6C). These results indicate that the G-to-A mutation does not completely abolish splice-site recognition, allowing a portion of transcripts to undergo normal splicing. To quantify splicing efficiency, band intensities were analyzed using ImageJ (Win64.exe). In *bmr34*, the normally spliced transcript accounted for approximately 36.2% of total transcripts, while the aberrantly spliced form represented about 63.8% (Figure 6D). Collectively, these results demonstrate that the G-to-A substitution predominantly causes abnormal splicing but retains partial normal splicing activity, confirming *bmr34* as a weak allele of *Bmr6*.

## 4. Discussion

Sorghum is an important forage crop, and biomass accumulation is a key target trait in forage breeding programs. Previous studies have shown that brown midrib (*bmr*) mutants typically reduce lignin content and improve forage digestibility [29]; however, these benefits are often accompanied by penalties in biomass production. Consistent with this, we previously reported that a coding-region mutation in *Sobic.004G071000* resulted in a 26.9% reduction in biomass [30]. In the present study, we identified a brown midrib mutant, *bmr34*, which represents a new allelic variant of *Sobic.004G071000*. The *bmr34* mutant exhibited typical brown midrib characteristics but did not show significant penalties in major agronomic traits, particularly biomass. Sequence alignment revealed a G-to-A single-nucleotide substitution at the 5′ splice site of the third intron in *Sobic.004G071000*. This mutation disrupted the conserved “GT–AG” splicing signal and triggered aberrant pre-mRNA splicing, resulting in the retention of a 33-bp fragment from the third intron within the third exon. The insertion introduced a premature stop codon (TGA), leading to early translational termination and the production of a truncated, nonfunctional protein. Notably, transcript analysis revealed that approximately 36.20% of *bmr34* transcripts were identical to the wild-type transcripts, indicating partial preservation of normal splicing. This residual production of functional transcripts likely mitigates the negative effects on biomass accumulation, providing a plausible explanation for the absence of biomass penalty in *bmr34*. Together, these findings suggest that *bmr34* represents a weak *bmr6* allele that balances improved forage quality with maintained biomass, highlighting its potential value for forage sorghum breeding.

Elucidation of the aberrant splicing mechanism in the *bmr34* mutant also provides new empirical evidence for understanding the complexity of pre-mRNA splicing regulation in eukaryotes. In eukaryotic genes, intron splicing depends on conserved cis-acting signals, among which the “GT–AG” rule is considered a core feature: the 5′ splice donor site is typically GT, whereas the 3′ splice acceptor site is AG. This rule is highly conserved across both plant and animal kingdoms [31]. In this study, mutation of the 5′ splice donor site of the third intron in *Sobic.004G071000* from GT to AT did not completely abolish intron removal. Instead, a subset of transcripts retained correct splicing and produced functional mRNA. This observation indicates that disruption of the canonical “GT–AG” motif does not necessarily result in complete splicing failure, highlighting a degree of flexibility in splice-site recognition. One possible explanation is that the assembly of a stable spliceosome complex partially compensates for the loss of splicing efficiency caused by the core nucleotide substitution, thereby permitting non-canonical splicing at this locus. It should be noted that this study focused exclusively on the third intron of *Bmr6*. Whether the other introns of this gene exhibit similar tolerance to splice-site mutations remains unknown. Consequently, the generality and locus specificity of this non-canonical splicing phenomenon require further investigation.

In recent years, CRISPR/Cas9 [32] has been widely applied in functional genomics [33] and crop improvement [34]. Most studies have focused on gene knockout or precise editing within coding regions [35], while a smaller number have targeted promoter regions to fine-tune gene expression [36], particularly in rice [37]. Moreover, recent advances in CRISPR gene-editing technology, such as the development and application of base editing and prime editing, have facilitated the expansion of targeted modification studies on cis-elements in promoters from rice to more cereal crops. In contrast, editing of splice sites has been rarely explored, largely due to limited understanding of splicing regulation and concerns about unpredictable effects. Our identification of the *bmr34* allele demonstrates that splice-site variation can generate partial-loss-of-function alleles with attenuated phenotypic effects. This finding suggests that targeted editing of splice sites represents a feasible strategy to modulate gene function rather than completely abolish it. Such an approach may be especially valuable for genes whose complete loss of function causes undesirable agronomic penalties. Therefore, splice-site editing could provide an alternative and potentially more precise strategy for functional modification of *Bmr6* and similar genes in sorghum breeding.

## Data Availability

The original contributions presented in the study are included in the article/Supplementary Materials. Further inquiries can be directed to the corresponding authors.

## Supplementary Materials

The following supporting information can be downloaded at: https://www.mdpi.com/article/10.3390/plants15111630/s1, Table S1: Primers used in the article; Table S2: Segregation analysis of the F_2_ population for the *bmr34* trait; Table S3: Statistical Table of Transcriptome Sequencing Results; Table S4: Brown midrib candidate gene annotation table.
